# C5b9 Deposition in Glomerular Capillaries Is Associated With Poor Kidney Allograft Survival in Antibody-Mediated Rejection

**DOI:** 10.3389/fimmu.2019.00235

**Published:** 2019-03-08

**Authors:** Valentin Goutaudier, Hélène Perrochia, Simon Mucha, Marie Bonnet, Sylvie Delmas, Florian Garo, Valérie Garrigue, Sébastien Lepreux, Vincent Pernin, Jean-Emmanuel Serre, Ilan Szwarc, Pierre Merville, Annie Ramounau-Pigot, Céline René, Jonathan Visentin, Bryan Paul Morgan, Véronique Frémeaux-Bacchi, Georges Mourad, Lionel Couzi, Moglie Le Quintrec

**Affiliations:** ^1^University of Montpellier, Department of Nephrology, Dialysis and Transplantation, Lapeyronie Hospital, Montpellier University Hospital, Montpellier, France; ^2^Department of Pathology, Gui de Chauliac Hospital, Montpellier University Hospital, Montpellier, France; ^3^Department of Nephrology, Transplantation, Dialysis and Apheresis, Pellegrin Hospital, Bordeaux University Hospital, Bordeaux, France; ^4^Department of Pathology, Pellegrin Hospital, Bordeaux University Hospital, Bordeaux, France; ^5^INSERM U1183, Institute for Regenerative Medicine and Biotherapy, Saint-Eloi Hospital, Montpellier University Hospital, Montpellier, France; ^6^UMR CNRS 5164, ImmunoConcEpT, Bordeaux University, Bordeaux, France; ^7^Department of Immunology, Saint Eloi Hospital, Montpellier University Hospital, Montpellier, France; ^8^Department of Immunology and Immunogenetics, Pellegrin Hospital, Bordeaux University Hospital, Bordeaux, France; ^9^School of Medicine, Systems Immunity Research Institute, Cardiff University, Cardiff, United Kingdom; ^10^Department of Immunology, Hospital European Georges Pompidou, Paris, France

**Keywords:** antibody-mediated rejection, kidney transplantation, complement, C4d, C5b9

## Abstract

C4d deposition in peritubular capillaries (PTC) reflects complement activation in antibody-mediated rejection (ABMR) of kidney allograft. However, its association with allograft survival is controversial. We hypothesized that capillary deposition of C5b9—indicative of complement-mediated injury—is a severity marker of ABMR. This pilot study aimed to determine the frequency, location and prognostic impact of these deposits in ABMR. We retrospectively selected patients diagnosed with ABMR in two French transplantation centers from January 2005 to December 2014 and performed C4d and C5b9 staining by immunohistochemistry. Fifty-four patients were included. Median follow-up was 52.5 (34.25–73.5) months. Thirteen patients (24%) had C5b9 deposits along glomerular capillaries (GC). Among these, seven (54%) had a global and diffuse staining pattern. Twelve of the C5b9+ patients also had deposition of C4d in GC and PTC. C4d deposits along GC and PTC were not associated with death-censored allograft survival (*p* = 0.42 and 0.69, respectively). However, death-censored allograft survival was significantly lower in patients with global and diffuse deposition of C5b9 in GC than those with a segmental pattern or no deposition (median survival after ABMR diagnosis, 6 months, 40.5 months and 44 months, respectively; *p* = 0.015). Double contour of glomerular basement membrane was diagnosed earlier after transplantation in C5b9+ ABMR than in C5b9– ABMR (median time after transplantation, 28 vs. 85 months; *p* = 0.058). In conclusion, we identified a new pattern of C5b9+ ABMR, associated with early onset of glomerular basement membrane duplication and poor allograft survival. Complement inhibitors might be a therapeutic option for this subgroup of patients.

## Introduction

Antibody-mediated rejection (ABMR) is the most common cause of allograft failure after kidney transplantation (1). In active ABMR, donor-specific antibodies (DSA) bind to graft endothelium, and activate complement-dependent and -independent mechanisms that recruit natural killer (NK) cells, neutrophils and macrophages which contribute to inflammatory lesions [peritubular capillaritis (PTC), glomerulitis], cellular necrosis and thrombotic microangiopathy. In chronic active ABMR, a repetitive pattern of thrombotic events and inflammatory changes result in endothelial cell injury and allograft matrix remodeling, such as transplant glomerulopathy (2). The complement system plays a key role in the pathophysiology of ABMR. C4d accumulation along PTC—which reflects the ability of the DSA bound to the surface of endothelial cells to activate the classical complement pathway—is recognized as a tissue footprint marker in ABMR (3, 4). Several *in vitro* assays have recently been developed to test the ability of DSA to bind complement products. Loupy et al. (5) demonstrated that positive C1q-binding DSA in the first year after transplantation was associated with poor graft survival. Sicard et al. (6) observed that positive C3d-binding DSA at the time of ABMR diagnosis was an independent risk factor for graft loss. Moreover, Lefaucheur et al. (7) showed that ABMR in patients with predominant DSA IgG3 subclass—which is the most able to activate the complement cascade—was associated with the poorest graft survival.

However, the *in vitro* complement-fixing ability of DSA does not reflect complement activation on the endothelial cell surface and the association between positive C4d staining with allograft survival remains controversial (8–11). They both do not indicate ongoing complement-mediated endothelial injury. Complement regulatory proteins can stop at any step the complement activation cascade on endothelial cell surface.

In contrast, the deposition of the C5b9 membrane attack complex indicates complete complement cascade activation. The terminal pathway directly activates endothelial cells through sublytic concentrations of C5b9 and/or recruitment of inflammatory cells by the anaphylatoxins C3a and C5a, and can also be responsible for endothelial cell lysis (1). However, in spite of the major role the C5b9 membrane attack complex plays in this damage, it has never been evaluated *in vivo* in kidney allografts.

This study aimed to determine the frequency and location of C5b9 deposits in a well-phenotyped cohort of patients experiencing ABMR, and to evaluate their impact on allograft survival.

## Methods

### Patients and Samples

We retrospectively selected transplant recipients with ABMR from the databases of the Departments of Pathology of two French University Hospitals (Montpellier and Bordeaux). To be included, patients had to be over 18 years and have undergone a renal biopsy that fulfilled criteria for a first histological diagnosis of (acute or chronic active) ABMR according to Banff 2015 classification from January 2008 to December 2013 at Montpellier Hospital and from January 2005 to December 2014 at Bordeaux Hospital, with positive DSA at time of biopsy. All biopsies were performed for cause: elevation of serum creatinine (>20% compared to baseline value) and/or a urine protein-to-creatinine ratio >50 mg/mmol. Complete immunofluorescence with anti-IgA, -IgG, -IgM, -C3, -C1q, -Kappa and -Lambda on frozen sections was performed in all patients. Exclusion criteria were the following: no serological evidence of anti-HLA DSA, insufficient renal tissue sample for further immunohistochemistry (i.e., <2 non-sclerosed glomeruli in each recut section), ABO-incompatible transplantation, combined transplantation, thrombotic microangiopathy and concomitant recurrent or *de novo* glomerulonephritis. The Institutional Review Board of Montpellier University Hospital approved this study (approval number: DC-2015-2473). All patients provided written informed consent to participate.

### Immunohistochemical Staining for C4d and C5b9

Staining for C4d and C5b9 was performed for all biopsies by immunohistochemistry. Briefly, paraffin-embedded sections were retrieved and cut at a thickness of 3-μm, deparaffinized and subjected to antigen retrieval. After blocking endogenous peroxidases, the sections were incubated with the relevant primary antibody. Binding of the primary antibody was visualized using the appropriate horseradish peroxidase-labeled secondary antibody and diaminobenzidine as the chromogen. Finally, the sections were counterstained with hematoxylin. The primary antibodies included rabbit monoclonal anti-human C4d (DB107, clone A24-T, dilution 1/100; DB Biotech) and mouse monoclonal anti-human C9 neoepitope (clone B7, dilution 1/50000; gift from Paul Morgan, Cardiff, United Kingdom), which is very specific to C5b9 fixation in membrane. C9 neoepitope detection was enhanced by the EnVision FLEX kit with linker (Dako).

The optimum antibody dilution and incubation were determined empirically for each primary antibody by performing a titration experiment and serial dilutions on positive (class 4 lupus nephritis) and negative controls (peritumoral kidney tissue).

The stained sections were evaluated by two renal pathologists who were blinded to the clinical and serological data. The sections were scored according to the presence of complement deposits along the PTC and glomerular capillaries (GC) of non-sclerosed glomeruli. Complement deposits in the glomerular mesangium and at the vascular pole were not taken into account. C4d and C5b9 deposition in the PTC were scored as minimal (>0 but <10% of PTC), focal (10–50% of PTC) or diffuse (>50% of PTC). C4d and C5b9 deposition in the GC were scored as segmental (<50% of capillary loops of all affected glomeruli) or global (≥50% of the capillary loops of at least 1 glomerulus) and focal (at least 1 glomerulus but <50% of glomeruli) or diffuse (≥50% of glomeruli). The staining intensity was evaluated using a semi-quantitative scoring system (negative = 0, weak = +, moderate = ++ and strong = + + +).

### Detection of Donor-Specific Antibodies

All patients were tested for circulating donor-specific anti-HLA -A, -B, -Cw, -DR, -DQA1, -DQB1, and -DP antibodies in serum samples obtained at the time of biopsy using single antigen flow bead assays (One Lambda) on a Luminex platform. All beads showing a normalized mean fluorescence intensity >500 were considered positive.

### Statistical Analysis

Quantitative variables were expressed as a mean with standard deviation or median with first and third quartiles. Qualitative variables were expressed by counts and percentages. We compared means, medians and percentages using the Student's *t*-test (or Wilcoxon test when appropriate) and chi-square test with Yates's correction (or Fisher's exact test when appropriate). Kidney allograft survival was assessed by the Kaplan-Meier method and compared among the groups with the log-rank test. Kidney allograft survival was calculated from the date of the ABMR-defining biopsy to the date of allograft loss (return to dialysis or new transplantation). If a patient died with a functioning graft, graft survival was censored at the time of death. Cox proportional-hazards models were used to estimate the hazard ratios (HR) and 95% confidence intervals (CI) for death-censored allograft loss. The association of clinical, functional, immunological and histological parameters with allograft loss was assessed with univariate Cox regression analyses. A *P-*value < 0.05 was used to select variables that were then entered into a single multivariate Cox model with stepwise backward elimination. R software (version 3.2.0) was used to perform all analyses. All tests were two-sided and *P-*values < 0.05 were considered statistically significant.

## Results

### Patient Characteristics

**Figure 1:**
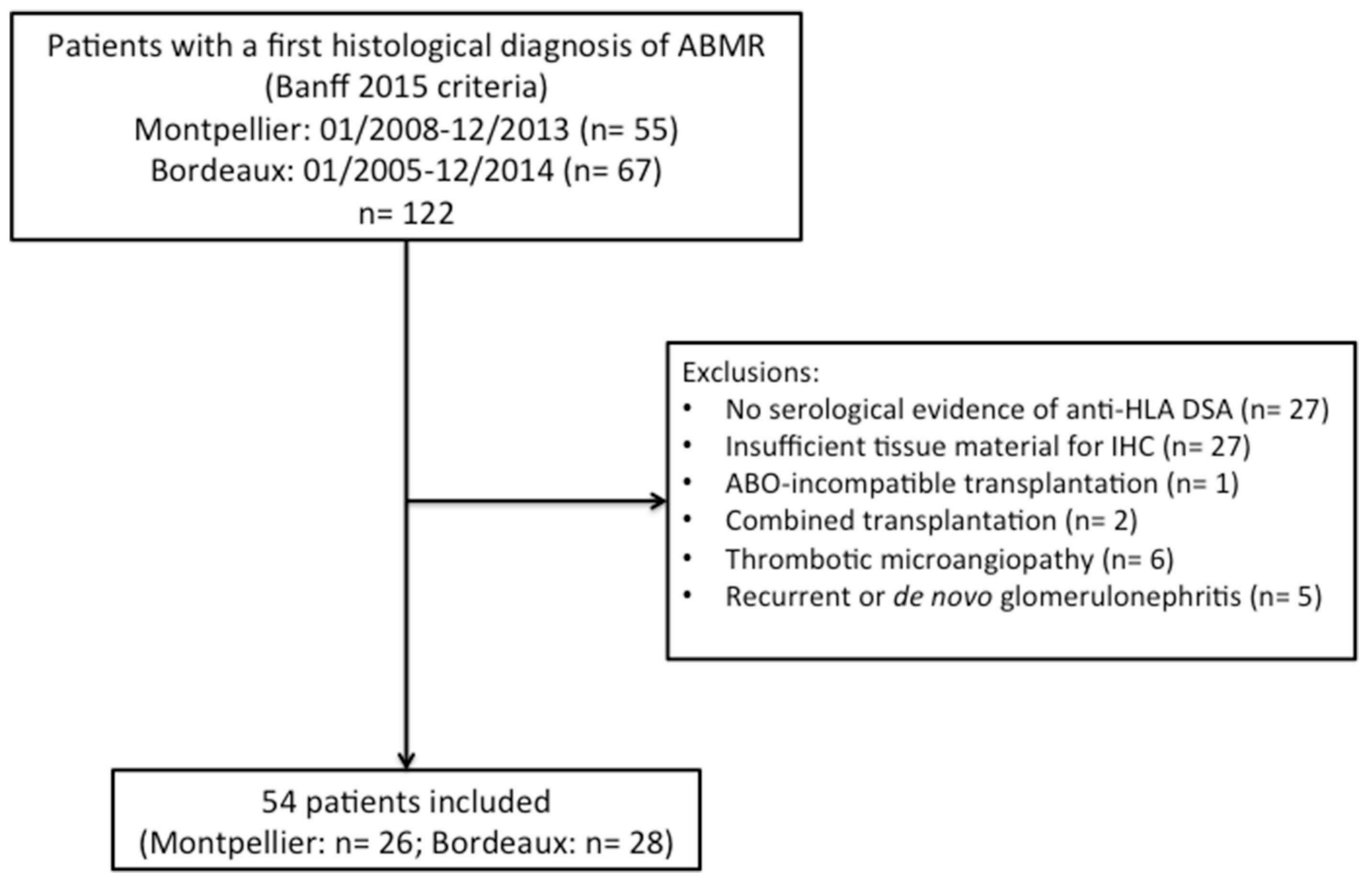
Flow chart. ABMR, antibody-mediated rejection; HLA, human leukocyte antigen; DSA, donor-specific antibody; IHC, immunohistochemistry.

During the study period, 122 patients (55 at Montpellier Hospital and 67 at Bordeaux Hospital) had a first histological diagnosis of ABMR of the current graft according to Banff 2015 criteria (4). Among them, 68 were excluded for the following reasons: no serological evidence of anti-HLA DSA (*n* = 27), insufficient tissue material for C4d and C5b9 detection by immunohistochemistry (*n* = 27), ABO-incompatible transplantation (*n* = 1), combined transplantation (*n* = 2), thrombotic microangiopathy (*n* = 6) and concomitant recurrent or *de novo* glomerulonephritis (*n* = 5). Finally, 54 patients were included (Figure 1), with a median follow-up of 52.5 months (range, 34.25–73.5 months). Their characteristics are summarized in Table 1. The median age of the patients was 49.5 years (35–59). Fifty patients (91%) had received their transplant from a deceased donor. Thirty-one patients (58%) had undergone an induction therapy with rabbit anti-thymocyte globulins. At the time of ABMR diagnosis, the mean estimated glomerular filtration rate was 34.31 ± 17.71 ml/min/1 m73^2^, and the median proteinuria level was 34 mg/mmol (19–121). Thirty-one (57%) patients had class I anti-HLA DSA; 47 (89%) had class II anti-HLA DSA; and 24 (45%) had class I and II anti-HLA DSAs. The immunodominant anti-HLA DSAs were class I in 12 (23%) patients and class II in 41 (77%) patients with a mean fluorescent intensity of 9000 (4365.5–14125).

**Table 1:** Patient characteristics.

**Variable**	***n***	**Value**
**Recipient characteristics**		
Age (years), median (Q1–Q3)	54	49.5 (35–59)
Male sex, *n* (%)	54	34 (63)
Nephropathy, *n* (%)	53	
Glomerulonephritis		21 (40)
Autosomal dominant polykystosis		6 (11)
Interstitial nephritis		3 (6)
Malformative uropathy		8 (15)
Others		5 (9)
Unknown		10 (19)
Blood group: A/B/AB/O, *n*	53	29/4/0/20
Time since dialysis (months), median (Q1–Q3)	50	30.5 (12–44.75)
**Transplant baseline characteristics**		
Preemptive, *n* (%)	52	4 (8)
Transplant number >1, *n* (%)	54	19 (35)
Deceased donor, *n* (%)	54	49 (91)
Expanded criteria donor, *n* (%)	54	16 (30)
Cold ischemia time (minutes), mean ± SD	50	1074.62 ± 540.78
Delayed graft function, *n* (%)	47	12 (26)
HLA A/B/DR mismatches, median (Q1–Q3)	52	4 (3–5)
**Immunosuppressive protocol**		
Induction therapy, *n* (%)	53	
Anti-thymocyte globulin		31 (58)
IL-2 receptor antagonist		21 (40)
Plasmapheresis		3 (6)
Intravenous immunoglobulin		6 (11)
Rituximab		3 (6)
Regimen at the time of biopsy	54	
None/ciclosporine/tacrolimus/mTORi, *n*		1/19/22/12
None/mycophenolic acid/azathioprine, *n*		5/46/3
Steroid withdrawal, *n*		12 (22)
Biology at the time of biopsy	54	
Creatininemia (μmol/l), median (Q1–Q3)	54	178 (140–223)
eGFR, MDRD formula (ml/min/1 m732), mean +/− SD	54	34.31 ± 17.71
Urine protein-to-creatinine ratio (mg/mmol), median (Q1–Q3)	45	34 (19–121)
**Characteristics of anti-HLA DSA at the time of biopsy**		
Class I, *n* (%)	54	31 (57)
Class II, *n* (%)	53	47 (89)
Class I + II, *n* (%)	53	24 (45)
**Immunodominant DSA**		
Class I, *n* (%)	53	12 (23)
Class II, *n* (%)	53	41 (77)
MFI, median (Q1–Q3)	52	9000 (4365.5–14125)
**Rejection treatment**		
Steroid pulses, *n* (%)	52	47 (90)
Switch to tacrolimus, *n* (%)	54	15 (28)
Plasmapheresis, *n* (%)	54	46 (85)
Intravenous immunoglobulin, *n* (%)	54	43 (80)
Rituximab, *n* (%)	52	30 (58)
Graft loss, *n* (%)	54	33 (61)
Death, *n* (%)	54	6 (11)
Follow-up (months), median (Q1–Q3)	54	52.5 (34.25–73.5)

*Q1, first quartile 1; Q3, third quartile; HLA, human leukocyte antigen; DSA, donor-specific antibody; MFI, mean fluorescence intensity; eGFR, estimated glomerular filtration rate; MDRD, modification of diet in renal disease; mTORi, mammalian target of rapamycine inhibitor*.

*Definitions: delayed graft function, dialysis within the first week after transplantation; expanded criteria donor, donor age > 60 years or 50 to 59 years with 2 of the following criteria: high blood pressure, cerebrovascular death cause, creatininemia > 132.6 μmol/l*.

### Histological Parameters of ABMR-Defining Biopsies

The median time of ABMR diagnosis after transplantation was 37.5 months (range, 6.25–76 months). Twenty-eight patients (52%) had acute ABMR and 26 (48%) had chronic active ABMR, with double contour of glomerular basement membrane. Nineteen patients (35%) had a concomitant T-cell mediated rejection. Positive C4d along PTC was found in 23/28 patients (82%) with acute ABMR and 21/26 (81%) with chronic active ABMR. Forty-five patients (85%) had interstitial fibrosis and tubular atrophy score ≥1. All histological parameters are listed in Table 2.

**Table 2:** Histological parameters of ABMR-defining biopsies.

**Variable**	***n***	**Value**
Time after transplantation (months), median (Q1–Q3)	54	37.5 (6.25–76)
Number of glomeruli, median (Q1–Q3)	54	10 (6–14)
Concomitant T-cell mediated rejection, *n* (%)	54	19 (35)
**Banff scores**		
Glomerulitis (g) score	54	
≥1, *n* (%)		34 (63)
Median (Q1–Q3)		1 (0–2)
Mesangial matrix expansion (mm) score	54	
≥1, *n* (%)		31 (57)
Median (Q1–Q3)		1 (0–1)
Double contour (cg) score	54	
≥1, *n* (%)		26 (48)
Median (Q1–Q3)		0 (0–2)
Interstitial fibrosis and tubular atrophy (IFTA) score	54	
≥1, *n* (%)		46 (85)
Median (Q1–Q3)		1 (1–2)
Interstitial inflammation (i) score	54	
≥1, *n* (%)		43 (80)
Median (Q1–Q3)		1 (1–2)
Tubulitis (t) score	54	
≥1, *n* (%)		19 (35)
Median (Q1–Q3)		0 (0–1)
Peritubular capillaritis (ptc) score	54	
≥1, *n* (%)		50 (93)
Median (Q1–Q3)		2 (1–2)
Arteriolar hyalinosis (ah) score	54	
≥1, *n* (%)		36 (67)
Median (Q1–Q3)		1 (0–1)
Vascular fibrous intimal thickening (cv) score	48	
≥1, *n* (%)		28 (58)
Median (Q1–Q3)		1 (0–1.25)
Vasculitis (v) score	48	
≥1, *n* (%)		6 (12)
Median (Q1–Q3)		0 (0–0)
Microvascular inflammation (g + ptc) score	54	
≥2, *n* (%)		41 (76)
Median (Q1–Q3)		2 (2–3)
C4d deposition in peritubular capillaries ≥2 (IF)	52	35 (67)

*ABMR, antibody-mediated rejection; Q1, first quartile; Q3, third quartile; IF, immunofluorescence*.

### Positivity and Location of C4d and C5b9 Deposits on ABMR Biopsies

**Figure 2:**
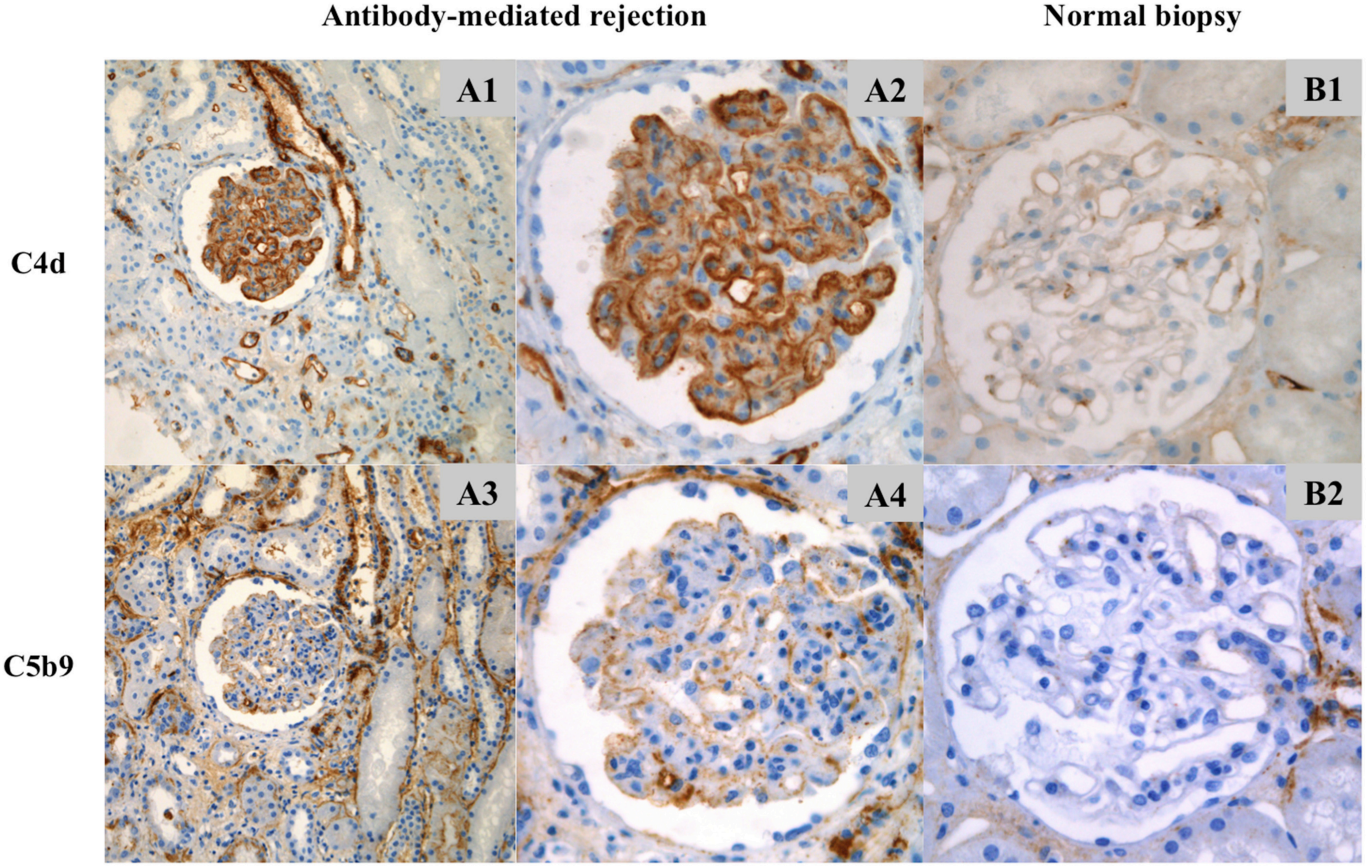
Location of C4d and C5b9 deposits by immunohistochemistry. **(A1–A4)** ABMR biopsy and **(B1,B2)** normal biopsy. **(A1)** C4d staining (x20), **(A2,B1)** C4d staining (x40), **(A3)** C5b9 staining (x20) and **(A4,B2)** C5b9 staining (x40). **(A1,A2)** In ABMR biopsy, C4d deposits were located in peritubular capillaries and along the glomerular capillaries (strong intensity), in the subendothelial space and/or intramembranous (pseudolinear pattern). **(A3,A4)** C5b9 staining was negative in peritubular capillaries and positive in glomerular capillaries (weak intensity), in the subendothelial space (granular pattern). There was no mesangial deposit. Positive staining in tubular basement membranes and Bowman's capsule are of non-specific significance. **(B1,B2)** C4d and C5b9 staining was negative in the normal biopsy. Arteriolar deposits are of non-specific significance.

The findings are summarized in Table 3. C4d was positive in PTC of 44/54 biopsies (81%) and in GC of 48/54 (89%). The deposition of C4d in PTC was minimal in 3/54 biopsies (6%), focal in 11/54 (20%), and diffuse in 30/54 (56%). The staining was weak in 3/44 patients (7%), moderate in 11/44 (25%) and strong in 30/44 (68%). Glomerular C4d deposition was pseudolinear and located in the subendothelial space and/or in the glomerular basement membrane (Figure 2). Deposition was segmental and focal in 4/54 biopsies (7%), segmental and diffuse in 3/54 (6%) and global and diffuse in 41/54 (76%). The staining was weak in 12/48 patients (25%), moderate in 12/48 (25%) and strong in 24/48 (50%).

**Table 3:** Location and intensity of C4d and C5b9 deposits by immunohistochemistry on ABMR biopsies (*n* = 54).

	**C4d**	**C5b9**
**Peritubular capillaries**, ***n*** **(%)**		
Positive staining	44 (81)	1 (2)
**Distribution**		
Negative	10 (19)	53 (98)
Minimal	3 (6)	0 (0)
Focal	11 (20)	1 (2)
Diffuse	30 (56)	0 (0)
**Intensity of positive Staining**		
Weak (+)	3 (7)	0 (0)
Moderate (++)	11 (25)	1 (100)
Strong (+++)	30 (68)	0 (0)
**Glomerular capillaries**, ***n*** **(%)**		
Positive staining	48 (89)	13 (24)
**Distribution**		
Negative	6 (11)	41 (76)
Segmental and focal	4 (7)	4 (7)
Segmental and diffuse	3 (6)	2 (4)
Global and diffuse	41 (76)	7 (13)
**Intensity of positive staining**		
Weak (+)	12 (25)	12 (92)
Moderate (++)	12 (25)	1 (8)
Strong (+++)	24 (50)	0 (0)

C5b9 was positive in PTC of one patient (1.8%) and in GC of 13/54 biopsies (24%). Glomerular C5b9 deposition was granular and subendothelial (Figure 2). Deposition was segmental and focal in 4/54 biopsies (7%), segmental and diffuse in 2/54 (4%) and global and diffuse in 7/54 (13%). Glomerular C5b9 staining was weak in 12/13 patients (92%) and moderate in one (8%). All but one of the biopsies with positive glomerular C5b9 deposition had C4d deposition in PTC (1 focal and 11 diffuse) and in GC (3 segmental and diffuse and 10 global and diffuse). Only one biopsy had C5b9 deposition in PTC associated with diffuse deposition of C4d in PTC and global and diffuse deposition of C4d and C5b9 in GC. Among the patients with global and diffuse deposition of C5b9 in GC, all except one had strong (+++) C4d staining along GC and PTC (Figure S1).

### Death-Censored Allograft Survival After ABMR Diagnosis According to the Deposition of C4d and C5b9

During a median follow-up of 52.5 months (range, 34.25–73.5 months), 33 patients (61%) lost their graft. Overall median death-censored allograft survival after ABMR diagnosis was 43 months (range, 29–59 months). Graft survival rates were respectively 83.3, 75.9, 70.1, and 27.2% at 3 months, 1 year, 2 years, and 5 years.

**Figure 3:**
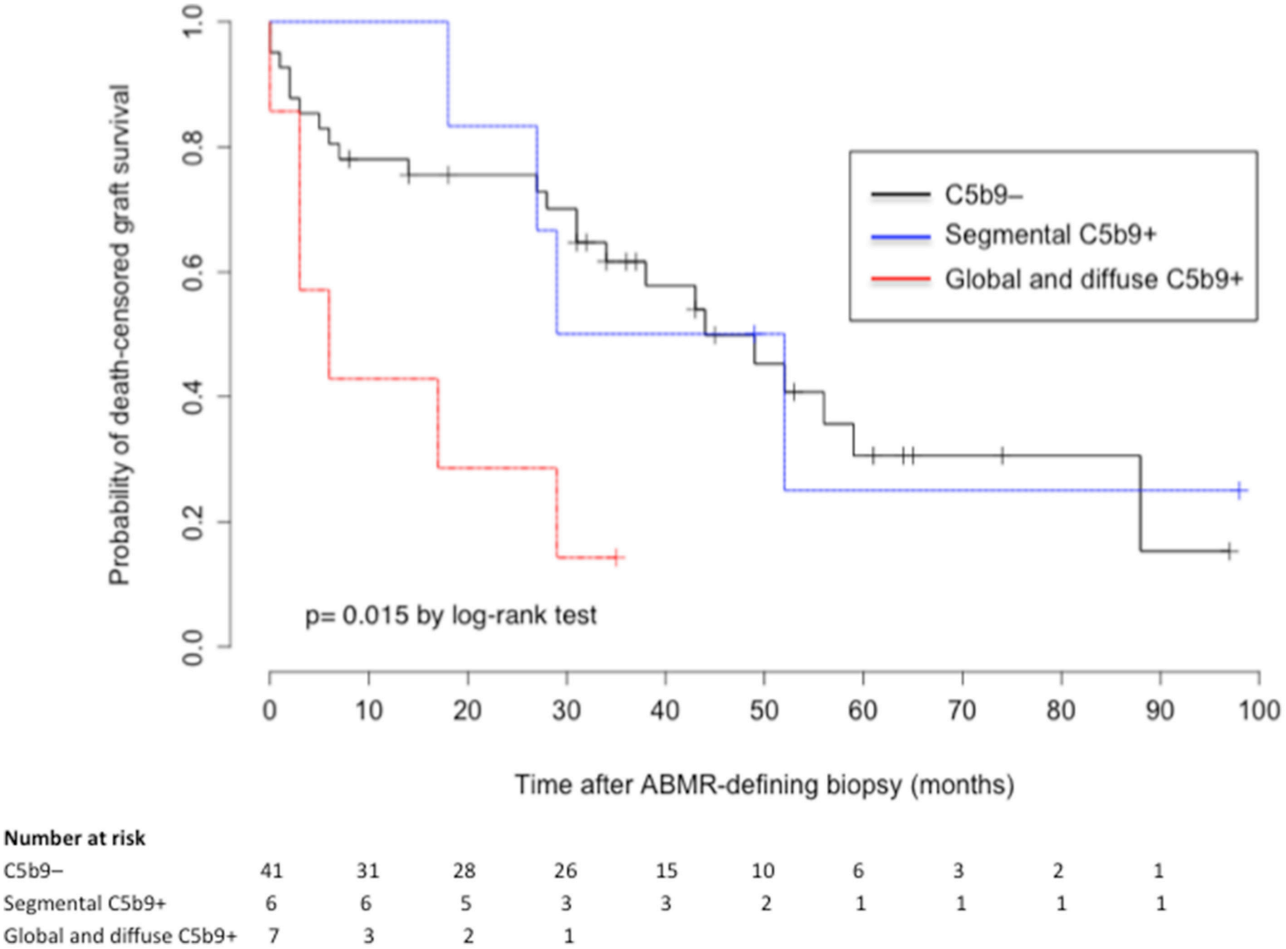
Death-censored graft survival after ABMR diagnosis according to the deposition of C5b9 in glomerular capillaries. ABMR, antibody-mediated rejection; segmental C5b9+, segmental (focal or diffuse) deposition of C5b9 in glomerular capillaries; global and diffuse C5b9+, global and diffuse deposition of C5b9 in glomerular capillaries.

C4d deposits in PTC and GC were not associated with allograft survival (*p* = 0.42 and 0.69 by log-rank-test, respectively). However, death-censored allograft survival was significantly lower in patients with global and diffuse deposition of C5b9 in GC than in those with a segmental pattern or no deposition (median survival after ABMR diagnosis: 6 months, 40.5 months, and 44 months respectively; *p* = 0.015 by log-rank-test; Figure 3). Graft survival rates in patients with global and diffuse deposition of C5b9 in GC were respectively 57.1, 42.9, and 28.6% at 3 months, 1 year and 2 years, compared to 85.4, 78.1, and 75.6% in patients without C5b9 deposit.

In a univariate Cox regression model (Table S1), the HR for death-censored allograft loss was 3.35 (95% CI, 1.46 to 9.60, *p* = 0.006) in patients with global and diffuse deposition of C5b9 in GC. In a multivariate analysis, this association was not found and the only independent risk factor for graft loss was the presence of double contour of glomerular basement membrane (adjusted HR 2.28, 95% CI 1.08 to 4.81, *p* = 0.03; Table S2).

### Phenotype of ABMR With Global and Diffuse Deposition of C5b9 in Glomerular Capillaries

**Figure 4:**
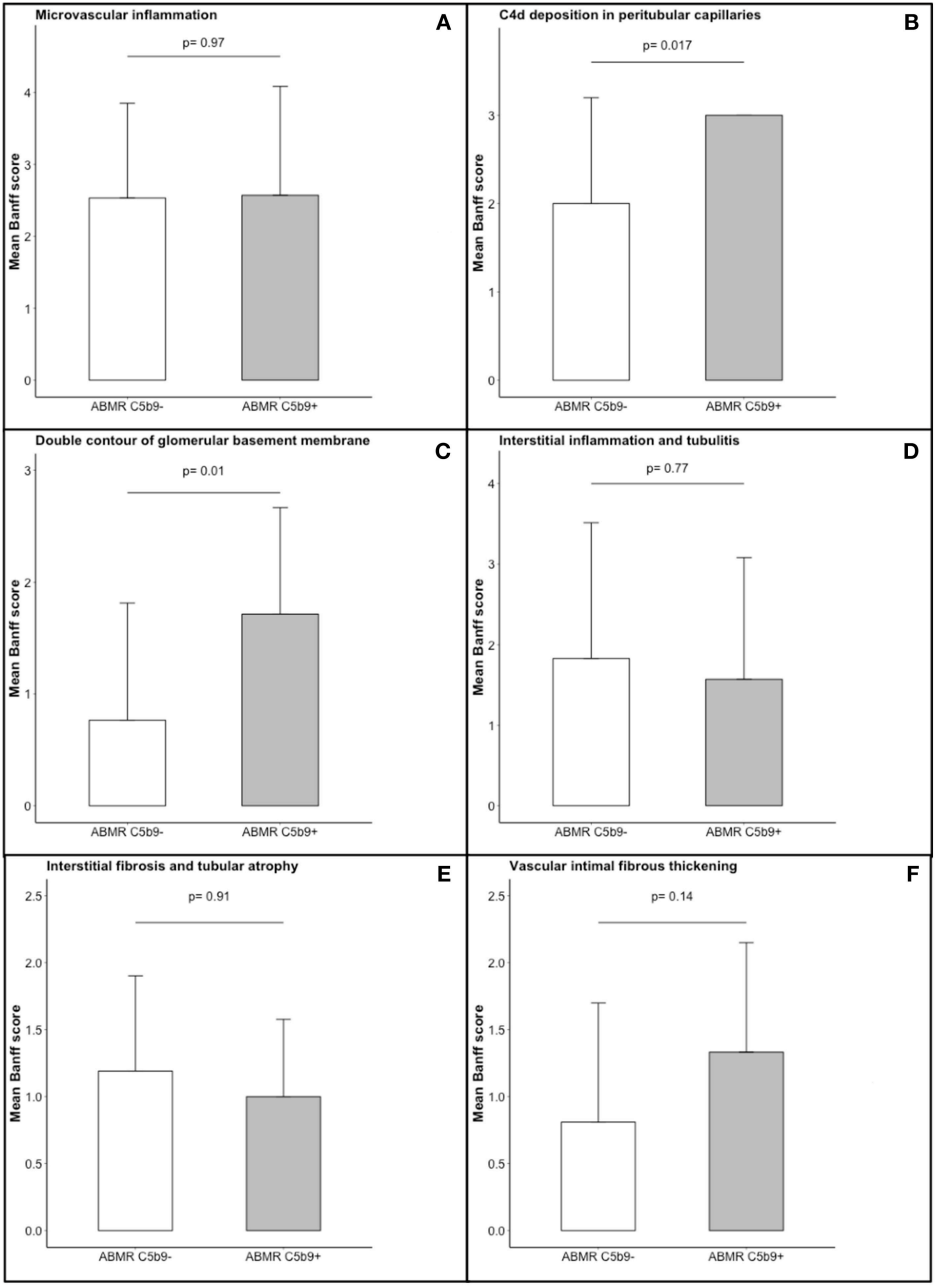
Comparison of histogical features in the two patterns of antibody-mediated rejection. The following Banff scores were compared: **(A)** microvascular inflammation [sum of the Banff scores for glomerulitis and peritubular capillaritis], **(B)** C4d deposition in peritubular capillaritis, **(C)** double contour of glomerular basement membrane, **(D)** interstitial inflammation and tubulitis, **(E)** interstitial fibrosis and tubular atrophy, and **(F)** vascular intimal fibrous thickening. Each of these scores ranges from 0 to 3, with higher score indicating more severe abnormality. The T bars indicate standard errors. ABMR C5b9−, antibody-mediated rejection without global and diffuse deposition of C5b9 in glomerular capillaries; ABMR C5b9+, antibody-mediated rejection with global and diffuse deposition of C5b9 in glomerular capillaries.

**Figure 5:**
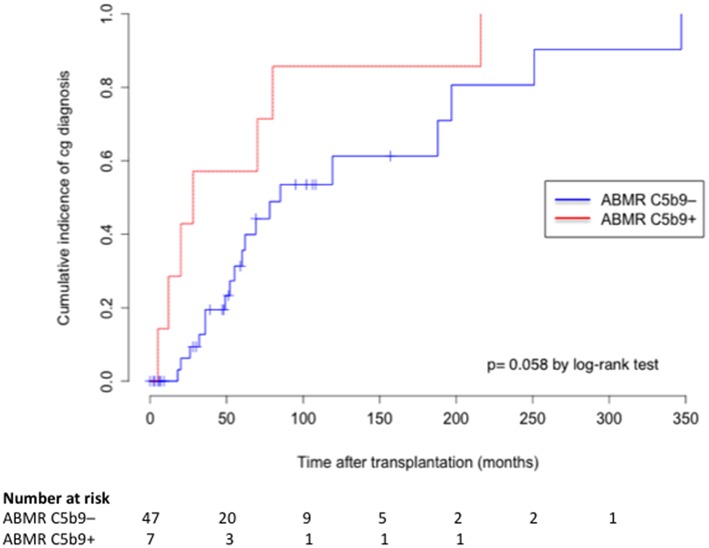
Cumulative incidence of cg diagnosis after transplantation according to the deposition of C5b9 in glomerular capillaries. cg, double contour of glomerular basement membrane; ABMR C5b9–, antibody-mediated rejection without global and diffuse deposition of C5b9 in glomerular capillaries; ABMR C5b9+, antibody-mediated rejection with global and diffuse deposition of C5b9 in glomerular capillaries.

Comparison of C5b9+ and C5b9– ABMR phenotypes is detailed in Table 4. The time to occurrence of ABMR after transplantation was not different between C5b9+ and C5b9– ABMR [28 (16–75) months vs. 39 (5.5–73.5) months, *p* = 0.75]. C5b9+ ABMR was associated with a worse graft function at diagnosis (median estimated glomerular filtration rate, 26 vs. 34 ml/min/1 m73^2^, *p* = 0.04) and tended to be associated with more anti-HLA DSA [3 (2–3.5) vs. 2 (1–3), *p* = 0.09), without any other difference in DSA characteristics. The occurrence of *de novo* DSA was similar in C5b9+ ABMR to C5b9– ABMR [4/5 (80%) vs. 22/30 (73%), *p* = 1.00], in the population (*n* = 35) who had been screened for anti-HLA antibodies by solid-phase assay before transplantation. All ABMR with global and diffuse deposition of C5b9 in GC had diffuse deposition of C4d in PTC and global and diffuse deposition of C4d in GC. Moreover, all of them displayed double contour of glomerular basement membrane, with a higher mean Banff score at diagnosis than that found in C5b9– ABMR (1.71 ± 0.95 vs. 0.77 ± 1.05, *p* = 0.01; Figure 4). These double contours were diagnosed earlier after transplantation in C5b9+ ABMR than in C5b9– ABMR (median time after transplantation, 28 vs. 85 months; *p* = 0.058; Figure 5). Among the patients with double contours, those with global deposits of C5b9 along GC were associated with a trend toward a worse death-censored allograft survival than the others (median survival after ABMR diagnosis, 6 vs. 29 months, *p* = 0.078 by log-rank test; Figure S2).

**Table 4:** Comparison of functional, immunological and histological variables in the two patterns of antibody-mediated rejection.

**Variables**	***n***	**C5b9– ABMR (*n* = 47)**	C5b9+ ABMR^*^ (*n* = 7)	***P-*value**
Time after transplantation (months), median (Q1–Q3)	54	39 (5.5–73.5)	28 (16–75)	0.75
**Functional (Biology at the Time Of Biopsy)**				
Creatininemia (μmol/l), median (Q1–Q3)	51	169 (139–212)	258 (197–304)	0.07
eGFR, MDRD formula (ml/min/1 m732), median (Q1–Q3)	54	34 (27–45.5)	26 (13.5–31)	0.04
Urine protein-to-creatinine ratio (mg/mmol), median (Q1–Q3)	45	34 (19–121)	58 (25.5–210)	0.78
**Immunology**				
DSA				
Number, median (Q1–Q3)	52	2 (1–3)	3 (2–3.5)	0.09
Class I, *n* (%)	53	25 (53)	6 (86)	0.22
Class II, (*n*%)	53	41 (89)	6 (86)	1.00
Class I + II, *n* (%)	53	19 (41)	5 (71)	0.28
Immunodominant DSA				
Class I, *n* (%)	53	10 (22)	2 (29)	0.65
Class II, *n* (%)	53	36 (78)	5 (71)	0.65
MFI, median (Q1–Q3)	52	9000 (3500–14000)	10335 (7142–13446.5)	0.52
**Histology**				
Concomitant TCMR, n (%)	54	17 (36)	2 (29)	1.00
Glomerulitis (g) score	54			
≥1, *n* (%)		30 (64)	4 (57)	1.00
Median score (Q1–Q3)		1 (0–2)	1 (0–1.5)	0.92
Mesangial matrix expansion (mm) score	54			
≥1, *n* (%)		25 (53)	6 (86)	0.22
Median score (Q1–Q3)		1 (0–1)	1 (1–2)	0.13
Double contour (cg) score	54			
≥1, *n* (%)		19 (40)	7 (100)	0.01
Median score (Q1–Q3)		0 (0–2)	1 (1–2.5)	0.01
Score, *n* (%)				0.002
0		28 (60)	0 (0)	
1		6 (13)	4 (57)	
2		9 (19)	1 (14)	
3		4 (9)	2 (29)	
Interstitial fibrosis and tubular atrophy (IFTA) score	54			
≥1, *n* (%)		40 (85)	6 (86)	1.00
Median score (Q1–Q3)		1 (1–2)	1 (1–1)	0.49
Interstitial inflammation (i) score	54			
≥1, *n* (%)		38 (81)	5 (71)	0.62
Median score (Q1–Q3)		1 (1–2)	1 (0.5–2)	0.91
Tubulitis (t) score	54			
≥1, *n* (%)		17 (36)	2 (29)	0.62
Median score (Q1–Q3)		0 (0–1)	0 (0−0.5)	0.63
Peritubular capillaritis (ptc) score	54			
≥1, *n* (%)		91% (43)	7 (100)	1.00
Median score (Q1–Q3)		2 (1–2)	2 (1–2)	0.99
Arteriolar hyalinosis (ah) score	54			
≥1, *n* (%)		30 (64)	6 (86)	0.40
Median score (Q1–Q3)		1 (0–1)	1 (1–1)	0.65
Vascular intimal fibrous thickening (cv) score	48			
≥1, *n* (%)		23 (55)	5 (83)	0.38
Median score (Q1–Q3)		1 (0–1)	1.5 (1–2)	0.14
Vasculitis (v) score	48			
≥1, *n* (%)		6 (14)	0 (0)	1.00
Median score (Q1–Q3)		0 (0–0)	0 (0–0)	0.39
Deposition of C4d in capillaries (IHC), *n* (%)	54			
Glomerular capillaries				0.85
No deposition		6 (13)	0 (0)	
Segmental and focal		4 (9)	0 (0)	
Segmental and diffuse		3 (6)	0 (0)	
Global and diffuse		34 (72)	7 (100)	
Peritubular capillaries				
None/minimal/focal/diffuse		10/3/11/23	0/0/0/7	0.15
C5b9 deposition in peritubular capillaries (IHC)		0 (0)	1 (14)	0.13

* *C5b9+ ABMR, antibody-mediated rejection with global and diffuse deposition of C5b9 in glomerular capillaries*.

*Q1, first quartile; Q3, third quartile; eGFR, estimated glomerular filtration rate; MDRD, modification of diet in renal disease; DSA, donor-specific antibody; MFI, mean fluorescence intensity; TCMR, T-cell mediated rejection; IHC, immunohistochemistry*.

## Discussion

This is the first study to evaluate C5b9 deposits in human kidney allografts with ABMR. In a cohort of 54 well-phenotyped kidney transplant recipients with ABMR from two centers, we observed that these deposits were present in a subgroup of patients, mainly located in the glomeruli and always associated with C4d deposits. ABMR with global and diffuse deposition of C5b9 in GC constituted a severe phenotype, with worse graft function at diagnosis and poor allograft survival. C5b9+ ABMR seems to be associated with early onset of glomerular basement membrane duplication and accelerated development of chronic glomerular lesions.

C4d deposition in PTC—a marker of active ABMR—is a footprint of the activation of the classical complement pathway after the binding of DSA to mismatched endothelial antigens (3, 4). In this study, about 80% of the patients had positive C4d in PTC and nearly 90% a pseudolinear deposition of C4d in GC. Interestingly, all the patients with C4d deposits along PTC had C4d in GC. The role of glomerular C4d in ABMR is not clearly established. The location of C4d deposits along glomerular endothelial cells, the negativity of C4d staining in T-cell mediated rejection and protocol graft biopsies (data not shown), and the strong association with positive C4d staining along PTC in our population supports the hypothesis that glomerular C4d plays a role in the pathogenesis of ABMR. Valente et al. (12) previously found similar results. In their study, glomerular C4d deposits along endothelial cells were present in cases of acute ABMR and correlated with C4d positivity along PTC. It is important to note here that in our study, patients were excluded if they had chronic/acute thrombotic microangiopathy associated with ABMR (13), immune complex-mediated glomerulonephritis and complement disease recurrence (14), because they are also associated with glomerular C4d positivity.

The endothelial C4d staining in ABMR reflects the first step of complement activation by the classical complement pathway. Although C4d staining in PTC is a helpful tool for diagnosing ABMR, its association with graft survival is controversial (10). In our study, C4d deposits along PTC and GC were not associated with graft survival. C4d is an inactive molecule, which has never been associated with endothelial injury *in vitro* and can also indicate a limited complement activation (15). The activation of the complement cascade is in fact tightly regulated by several fluid phase and membrane-bound proteins (16). Complete complement cascade activation up to the terminal pathway—expressed by positive C5b9 staining—can reflect overactivation of the complement system, defective complement regulatory proteins or an imbalance between activation and regulation. In our study, C5b9 deposits were positive in 24% of the ABMR biopsies, mainly along GC. All C5b9+ ABMR had positive C4d staining along PTC and GC indicating initiation by the classical complement pathway and full complement cascade activation. The fact that C5b9 was mainly positive in glomeruli and along with C4d deposits, supports the role of glomerular complement activation in the pathogenesis of ABMR. Of note, C5b9 is also often positive in glomeruli, but not in PTC, in thrombotic microangiopathy (13).

Global and diffuse glomerular deposition of C5b9—which reflects unregulated complement activation or a massive production of complement products due to an excessive complement activation—occurred in 13% of ABMR in our study, and was associated with massive C4d deposits along PTC and GC. This characterizes a severe phenotype, with poor graft function at diagnosis and poor allograft survival. A trend for a higher number of DSA was the only factor we found to explain this uncontrolled injury. However, endothelial susceptibility to injury cannot be excluded, maybe secondary to a lack of complement regulatory proteins. Indeed, patients with segmental deposition of C5b9 in GC—which reflects partially regulated complement activation—had a similar graft survival than patients without C5b9 deposits—which reflects regulated complement activation. Moreover, the absence of C5b9 deposits along PTC could suggest a better complement regulation or the inability to form a membrane complex attack in these small vessels. Finally, it is important to note that although glomerular C5b9 staining was often weak, it was very specific because we used a mouse monoclonal anti-human C9 neoepitope—which is specific to C5b9 fixation in membrane—and it was strictly negative in negative controls. Moreover, in opposition with the intensity of C4d staining, the weakness of C5b9 staining is probably the reflection of the regulation of complement activation by endothelial cells after the cleavage of C4.

Multivariate analysis did not confirm that global and diffuse deposition of C5b9 in glomerular capillaries was associated with poor graft survival. The only independent risk factor for graft loss was the presence of double contours of glomerular basement membrane, which was associated with more than a 2-fold risk of graft loss. This result could be explained by a lack of power (there was a trend toward a lower graft survival in cg+ C5b9+ ABMR than cg+ C5b9-) but also by the fact that all patients with global and diffuse C5b9 deposits had double contours. This lesion is widely accepted to be a major prognostic factor in ABMR, possibly the most important one (17, 18). The pathogenesis of transplant glomerulopathy—currently viewed as a cardinal histological lesion of chronic ABMR—remains unclear (19). Recently, Gasim et al. (20) found that pseudolinear GC C4d correlated with the severity of glomerular basement membrane duplication by light and electron microscopy. The authors hypothesized that complement activation could be involved in this multi-layering process. However, C5b9 staining was not performed in this study. In our cohort, we observed that 25/26 (96%) patients with double contours had C4d deposition in GC indicating complement activation. Strikingly, patients who also had global and diffuse C5b9 deposits had fast onset of double contours, suggesting devastating endothelial injury. C5b9 can activate endothelial cells at sublytic concentrations and induce a pro-inflammatory state, mainly through non-canonical nuclear factor-κB signaling (21, 22). Owing to the observational nature of this study, we cannot confirm a causal link between C5b9 and double contours. Nethertheless we hypothesize that C5b9 can activate glomerular endothelial cells and accelerate the duplication of glomerular basement membrane, because we diagnosed double contours earlier in C5b9+ ABMR than in C5b9- ABMR and there was a trend toward a worse death-censored allograft survival in C5b9+ ABMR among the patients with double contours This could be a result of repeated episodes of endothelial activation, injury, and repair (19). However, the duplication of glomerular basement membrane is not specific of chronic ABMR. It has been largely described in complement-mediated diseases such as chronic thrombotic microangiopathy and membranoproliferative glomerulonephritis (13, 14), which were excluded in our study.

It is important to note that C5b9 was positive in a subgroup of patients, indicating that complement activation is often efficiently regulated. Endothelial injury can also be secondary to another complement-mediated mechanism—the recruitment of inflammatory cells by the anaphylatoxins C3a and C5a—and/or to complement-independent mechanisms, such as antibody-dependent cell cytotoxicity and direct endothelial cell activation by the antigen-binding fragment of DSA (23). This relatively uncommon positivity of C5b9 could explain the conflicting results of anti-C5 therapies in the prevention and treatment of ABMR in unselected cohorts (24–26). To reassess the value of this therapy in ABMR, it could be helpful to select patients on the basis of demonstrated complement-mediated endothelial injury.

This study has limitations. Firstly, it was retrospective, and a significant number of patients were excluded for missing data (mostly insufficient tissue on recut biopsy sections). Secondly, we probably underestimated C4d-negative ABMR. This is because, prior to the Banff 2013 classification, C4d was mandatory for a diagnosis of ABMR (27). As our study inclusion period started in 2008, some cases of C4d-negative ABMR might not have been classified as ABMR and therefore not included. Thirdly, all biopsies were performed for cause, so we did not study subclinical ABMR, which is also closely associated with graft survival (28). Lastly, unfortunately we were not able to study ultrastructural changes of glomerular basement membrane by electronic microscopy, complement abnormalities nor complement-binding DSA abilities.

In conclusion, we have defined a new pattern of ABMR of renal allografts with complete complement cascade activation up to the terminal pathway in a subgroup of patients. This constitutes a severe phenotype, with diffuse deposits of C4d along PTC, C4d/C5b9 along GC, early onset of glomerular basement membrane duplication and poor allograft survival. It could be interesting to analyze C4d and C5b9 deposits in cases of subclinical ABMR to evaluate if their positivity precedes the occurrence of double contours. Moreover, the value of anti-C5 therapies in this specific subgroup of patients should be assessed.

## Author Contributions

The study was conceived and designed by VGo and ML. VGo and ML conducted analysis. VGo and ML were involved in the writing of the manuscript. VGo, ML, and all other authors contributed to the conduct of the study, recruited patients, and were involved in the review of results and final approval of the manuscript.

### Conflict of Interest Statement

The authors declare that the research was conducted in the absence of any commercial or financial relationships that could be construed as a potential conflict of interest.

## Abbreviations

ABMR: antibody-mediated rejection
C4d: complement split product C4d
C5b9: membrane attack complex
cg: double contour of glomerular basement membrane
DSA: donor-specific antibody
GC: glomerular capillaries
HLA: human leukocyte antigen
PTC: peritubular capillaries
TCMR: T cell-mediated rejection.
