# Supplementary material for: C5b9 Deposition in Glomerular Capillaries Is Associated With Poor Kidney Allograft Survival in Antibody-Mediated Rejection
Source: Front Immunol. 2019 Mar 8;10:235. doi: 10.3389/fimmu.2019.00235 (PMC6418012; doi:10.3389/fimmu.2019.00235)
Supplement: Supplementary file 1 [file Data_Sheet_1.docx]

**Supplementary material**


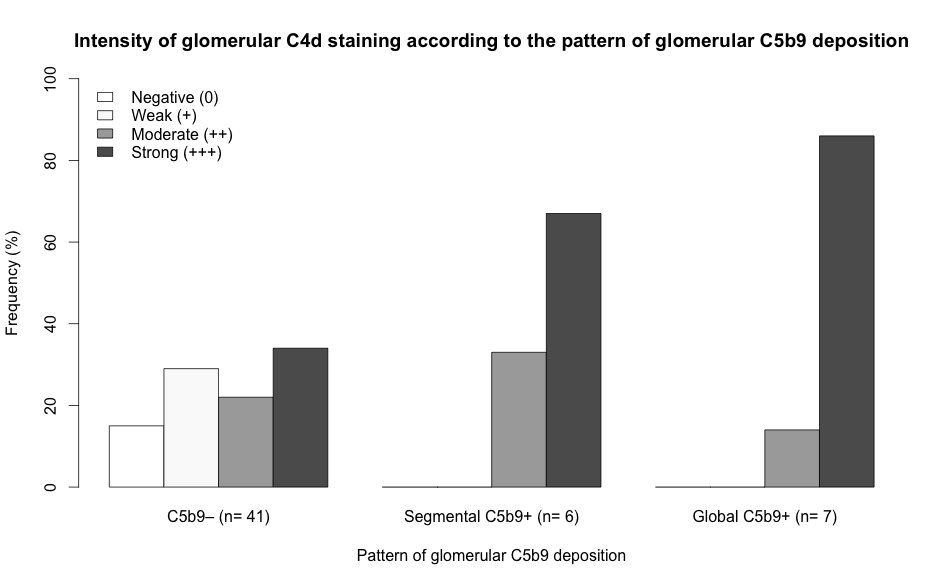


**Figure S1. Intensity of glomerular C4d staining according to the pattern of glomerular C5b9 deposition.**

C5b9–, negative C5b9 staining in glomerular capillaries; segmental C5b9+, C5b9 deposition in < 50% of capillary loops of all affected glomeruli; global C5b9+, C5b9 deposits in ≥ 50% of the capillary loops of at least 1 glomerulus.

**Figure S2. Death-censored graft survival after cABMR diagnosis according to the deposition of C5b9 in glomerular capillaries.**

cABMR, chronic antibody-mediated rejection with double contour of glomerular basement membrane; cABMR C5b9–, cABMR without global and diffuse deposition of C5b9 in glomerular capillaries; cABMR C5b9+, cABMR with global and diffuse deposition of C5b9 in glomerular capillaries.

**Table S1. Univariate analysis of variables associated with death-censored allograft loss.**

**Table S2. Multivariate analysis of variables associated with death-censored allograft loss.**
